# USING BEST PROGRAMS FOR CAREGIVING TO MEET THE DIVERSE NEEDS OF GUIDE MODEL SITES AND BENEFICIARIES

**DOI:** 10.1093/geroni/igae098.0035

**Published:** 2024-12-31

**Authors:** Morgan Minyo, Katie Maslow, David Bass, Rachel Cannon, Zoe Fete, Megan Huth, Sara Powers

**Affiliations:** Benjamin Rose Institute on Aging, Cleveland, Ohio, United States; Retired, Bethesda, Maryland, United States; Benjamin Rose, Cleveland, Ohio, United States; Benjamin Rose, Cleveland, Ohio, United States; Benjamin Rose, Cleveland, Ohio, United States; Benjamin Rose, Cleveland, Ohio, United States; Benjamin Rose, Cleveland, Ohio, United States

## Abstract

In 2023, the Center for Medicare and Medicaid Innovation announced a new alternative payment model for comprehensive dementia care called the Guiding an Improved Dementia Experience (GUIDE) Model. The GUIDE Model is intended to improve quality of life for people living with dementia (GUIDE Beneficiaries) and reduce strain on unpaid caregivers through a package of care coordination and management, caregiver education and services, and respite care. Health systems and other medical or community-based organizations that applied and were selected as GUIDE Model Sites must offer specific program components and care deliver requirements to identify services and supports needed by both Guide Beneficiaries and their caregivers. This requires GUIDE Model Sites to potentially add new supports to their dementia care or be able to link Beneficiaries and their caregivers to services that meet the diverse and changing needs of both individuals. The Best Programs for Caregiving (BPC) online database is a valuable resource that can benefit GUIDE Participating Sites and others, ensuring that family/friend caregivers are appropriately recognized and included within Beneficiary’s dementia care. The dementia support programs in BPC meet GUIDE Model care delivery requirements including one-on-one and group caregiver education, care coaching, and ongoing monitoring and support. Additionally, BPC programs have proven positive outcomes for dementia family/friend caregivers, are actively being delivered by organizations across the US, and describe if/how a program has been adapted or delivered to caregivers from diverse communities. BPC’s extensive database can help provide the necessary information for high-quality dementia care under the Guide Model.

